# Phytol-based novel adjuvants in vaccine formulation: 2. assessment of efficacy in the induction of protective immune responses to lethal bacterial infections in mice

**DOI:** 10.1186/1476-8518-4-5

**Published:** 2006-10-23

**Authors:** So-Yon Lim, Adam Bauermeister, Richard A Kjonaas, Swapan K Ghosh

**Affiliations:** 1 Department of Life Sciences, Indiana State University, Terre Haute, IN 47809, USA; 2 Department of Chemistry, Indiana State University, Terre Haute, IN 47809, USA

## Abstract

**Background:**

Adjuvants are known to significantly enhance vaccine efficacy. However, commercial adjuvants often have limited use because of toxicity in humans. The objective of this study was to determine the comparative effectiveness of a diterpene alcohol, phytol and its hydrogenated derivative PHIS-01, relative to incomplete Freund's adjuvant (IFA), a commonly used adjuvant in augmenting protective immunity in mice against *E. coli *and *S. aureus*, and in terms of inflammatory cytokines.

**Methods:**

Vaccines, consisting of heat-attenuated *E. coli *or *S. aureus *and either of the two phytol-based adjuvants or IFA, were tested in female BALB/c mice. The vaccines were administered intraperitoneally at 10-day intervals. The efficacy of the phytol and PHIS-01, as compared to IFA, was assessed by ELISA in terms of anti-bacterial antibody and inflammatory cytokines. We also examined the ability of the vaccines to induce specific protective immunity by challenging mice with different doses of live bacteria.

**Results and discussion:**

IFA, phytol, and PHIS-01 were equally efficient in evoking anti-*E. coli *antibody response and in providing protective immunity against live *E. coli *challenges. In contrast, the antibody response to *S. aureus *was significant when PHIS-01 was used as the adjuvant. However, in terms of the ability to induce protective immunity, phytol was most effective against *S. aureus*. Moreover, during challenges with live *E. coli *and *S. aureus *immune mice produced much less IL-6, the mediators of fatal septic shock syndromes.

**Conclusion:**

Our results show that vaccine formulations containing phytol and PHIS-01 as adjuvants confer a robust and protective immunity against both Gram-negative and Gram-positive bacteria without inducing adverse inflammatory cytokine due to IL-6.

## Background

Protective immunity in vertebrates depends largely on efficient activation and subsequent interactions of cells belonging to both innate and acquired immunity. The innate component, however, has no memory and is first to respond with only a limited repertoire to recognize pathogen-associated molecular patterns (PAMPs), usually present in the cell wall structures of microbes [1-3]. PAMPs function as immunoadjuvants or immunostimulants and up-regulate TOLL-like receptors of cells belonging to innate immunity that in turn activates the specific acquired immunity. [4,5]. The activation of specific immunity mediated by B and T lymphocytes requires at least 3–4 days, but it lasts much longer and responds specifically to a diverse range of microbial antigens [6,7]. Therefore, vaccines that interact with both components of the immune system have the ability to induce effective prophylaxis against a variety of infectious diseases.

In bacterial infection, antibody response is crucial for neutralizing bacterial toxins or blocking their attachment to host cells [8]. Antibodies also help recruit complement to kill and dispose of organisms, as well as enhance the binding and uptake by phagocytes [9]. Most anti-microbial vaccines in current use have been designed to stimulate antibody responses, and the development of effective cell-mediated immunity is also important to overcome chronic infectious diseases associated with intracellular pathogens and viruses [10]. In spite of historical successes with killed or attenuated microbes as vaccines, the looming threat of new and resistant pathogens requires development of new and improved versions of vaccines.

Molecular vaccines produced by modern technology (e.g. synthetic peptides, DNA vaccines) are safer than traditional vaccines composed of inactivated or killed organisms [11]. Because of a relatively limited antigenic repertoire, molecular vaccines are often poorly immunogenic and depend on the co-administration of immunostimulants or adjuvants to be effective. Therefore, there is a constant need to develop new adjuvants that are 1) safe for clinical use; 2) able to enhance immunogenicity of vaccine proper; and 3) likely to improve the performance of both traditional and molecularly defined vaccines. Unlike the vaccine itself, which is often restricted in action to specific and cross-reactive antigens, adjuvants have much wider usage as immunostimulants with many different vaccines. In our ongoing study [12], we have observed that phytol, an aliphatic alcohol component of chlorophyll, and its derivative, PHIS-01, are excellent adjuvants, superior in many respects to commonly used and commercially available standard adjuvants (Complete/Incomplete Freund's adjuvant, Alum, Titermax, and Ribi adjuvant system). Unlike the conventional adjuvants, these phytol-based adjuvants are safe with high benefit-to-toxicity ratio, generate more IgG2a-type complement-fixing antibodies, and trigger no lupus-like syndromes in susceptible mice [12]. They also augment cytotoxic T cell response (CTLs) directed toward a murine B-cell lymphoma [12].

This study was initiated to determine the effectiveness of phytol-based adjuvants against commonly occurring gram-negative and gram-positive bacteria. *Escherichia coli *and *Staphylococcus aureus *are prevalent pathogens associated with nosocomial and community-acquired infections of various body sites and disease processes [13,14]. We report here that phytol-based adjuvants enhance the immune responses to *E. coli *and *S. aureus *infections better than IFA, the commonly used commercial adjuvant with fewer side effects. Phytol-based adjuvants influence many parameters of immune response including its intensity, duration, isotype and specificity. Furthermore, immunizations in mice using whole, inactivated bacterial cells plus the phytol adjuvants provide a lasting protection against intraperitoneal challenge with high doses of live *E. coli*. Equally effective are heat-attenuated *S. aureus *vaccines plus phytol adjuvants, which produce excellent antibody response and lasting immunity to *S. aureus *challenge.

## Methods

### Bacterial culture

Bacteria, *E. coli *(ATCC number: 14948) and *S. aureus *(ATCC number: 25935,) kindly provided by Dr. H. K. Dannelly of the Department of Life Sciences), were cultured in LB broth (Difco, Detroit, MI) at 37°C for 14 hours and harvested with phosphate buffered saline (PBS). Cells were washed in PBS by centrifugation at 500 × g for 10 min at 4°C and then suspended to the appropriate density in PBS. Bacteria were killed by heating suspensions to 60°C for 1 hr.

### Preparation of immunogen

Test vaccines consisted of either 5 × 10^6 ^CFU of *E. coli *or *S. aureus *and various adjuvants in a total volume of 400 μl. Adjuvants used in this study include: IFA (Sigma Chemical Co., St. Louis, MO), phytol (Pfaltz and Bauer Inc., Waterbury, CT) and PHIS-01 (Patent pending). PHIS-01 is one of several chemically modified phytol-based adjuvants developed in this laboratory.

### Mice-immunization and challenge

Female BALB/c mice (6–8 weeks of age) were used for all experiments. These mice were bred and maintained at the animal care facility of Indiana State University. The use of these mice has been guided by strict adherence to an approved protocol prepared under the supervision and oversight of the Indiana State University Animal Care and Use Committee.

Immunization was carried out in groups of 4–5 mice using either 5 × 10^6 ^CFU of *E. coli *or *S. aureus *and test adjuvants (IFA, Phytol, or PHIS-01) in a total volume of 400 μL. Bacteria without adjuvant in a total volume of 400 μL PBS were used as control. Vaccines were administered intraperitoneally (I.P.) and animals were immunized three times at 10-day intervals. Sera were collected 5–7 days after each immunization for analysis by ELISA.

For the protection assay, mice were challenged with an IP injection of *E. coli *or *S. aureus *(10^6^, 10^7^, and 10^8 ^CFU/mouse) in 1.0 ml of PBS. Challenges took place on day 5 after the third immunization.

### Preparation of bacterial cell lysates

Bacterial cultures were harvested and washed in PBS. Cells were lysed in 1 ml of buffer containing 8 M urea, 0.01 M Na-phosphate (dibasic), 0.01 M Tris-HCl (pH 8.0), and 5 μl of protease inhibitor cocktail. Cell debris was removed by centrifugation at 13,000 × g for 5 min at 4°C. Supernatant was collected and protein concentration was estimated from absorbance at 280 nm. Adopting a procedure reported by Gomez *et al*. [6], these lysates were used to coat 96-well microtiter plates for ELISA.

### Enzyme-Linked Immunosorbent Assay (ELISA)

Antibody levels of mouse sera were measured routinely by a binding assay to antigen-coated ELISA plates:

(a) Antibody specific to bacterial strain: Cell-lysate coated ELISA plates were prepared by incubating polyvinyl 96-well plates with 10 μg/ml cell lysates in 0.01 M sodium bicarbonate solution overnight at 4°C. After blocking with 1% BSA/PBS overnight at 4°C, serially diluted sera obtained from immunized mice were added to each well, and incubated for 1 hr at 37°C. Plates were incubated with rabbit anti-mouse Ig-HRP and washed. Bound rabbit anti-mouse Ig-HRP was detected by addition of *o*-phenylene diamine (OPD), and the intensity was measured at 490 nm. Specific IgG antibodies were expressed as the mean ± SEM.

(b) Antibody specific to LPS: LPS (Sigma Chemical Co., St. Louis, MO) suspended in PBS were placed in poly-L-lysine (Sigma, St. Louis, MO) precoated ELISA plates and incubated for 1 hr at 37°C. The plates were washed three times with PBS containing 0.05% Triton-X, and ELISAs performed using conditions described by Takahashi et al [15].

### Antibody-subclass determination

To determine the characteristics of antibody response induced by vaccination, mouse immune sera were typed for IgM, IgG1, IgG2a, IgG2b, and IgG3 classes using anti-mouse Ig subclass-specific HRP-conjugated secondary antibodies (Zymed, San Francisco, CA), following the manufacturer's protocol. The ratio of IgG1 and IgG2a isotypes was calculated by dividing the A_405 _values for IgG1 by IgG2a.

### SDS-PAGE and Western blot

Cell lysates, as described above, were mixed with an equal volume of SDS-PAGE sample buffer (Bio-Rad Laboratories, Hercules, CA) and electrophoresed on 12% polyacrylamide gels. The proteins were transferred to nitrocellulose membranes and rabbit anti-mouse Ig (A + M + G) (ICN, Irvine, CA) antibodiesand HRP-conjugated goat anti-rabbit Ig (Sigma Chemical Co., St. Louis, MO) were used to detect total Ig (M + G). HRP-conjugated rabbit anti-mouse IgG (Sigma Chemical Co., St. Louis, MO) was used to detect specific IgG type antibodies. Color development was accomplished using Supersignal® chemiluminescent (Pierce, Rockford, IL).

### Cytokine assays

Proinflammatory cytokines, IL-6 and TNF-α levels in blood and peritoneal lavage were determined by ELISA. ELISA was performed in triplicate using specific mAbs (eBioscience, San Diego, CA) according to the manufacturer's instructions.

### Monitoring of infection and mortality

These studies were conducted in PBS-treated control and experimental mice in various groups. These mice were vaccinated with killed bacterial lysates in the presence or absence of adjuvants. Animal mortality was assessed every 12 h during the first 3 days following bacterial challenges (10^6^, 10^7 ^or 10^8 ^CFU/mouse) and peritoneal fluid and blood were cultured to confirm infection. Mortality occurred predominantly between 12 h and 36 h after challenge. At 18 h after bacterial challenge, blood and peritoneal lavages were obtained for quantitative culturing. Samples from all groups of mice were diluted in LB medium (1:200), and a 10 μl sample of each of these was streaked on agar plates using calibrated loops to detect bacteremia caused by >10^3 ^CFU/ml.

### Statistical analysis

Statistical analyses of all data were done by the paired Student's *t*-test (Sigma Plot). For all statistical tests, alpha was set at 0.05. All data were expressed as mean ± SEM in the figures and tables.

## Results

### Specific anti-bacterial antibody responses

**Figure 1:**
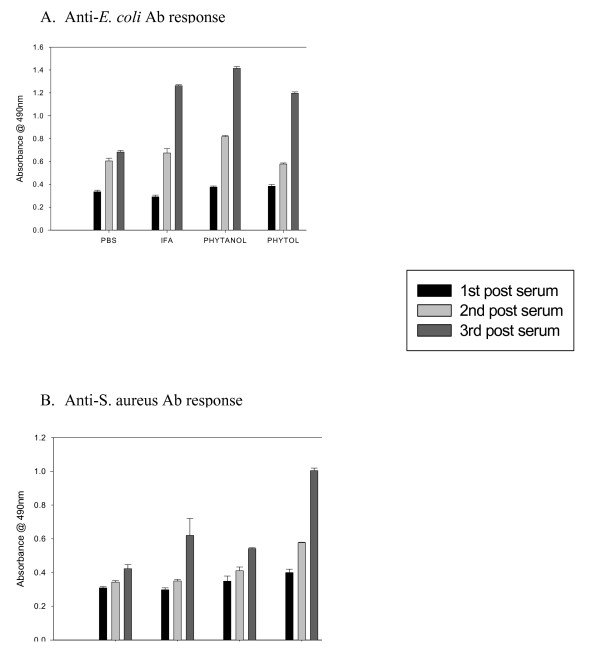
Antibody response in BALB/c mice following immunization with bacterial vaccines prepared in test adjuvants. Control mice were either unimmunized or immunized with vaccines in PBS with no adjuvant. The ΔOD^490 ^values were obtained by subtracting absorbance due to normal mouse sera from the experimental ones. Polyclonal antisera obtained from *E. coli *or *S. aureus *were evaluated at 1:200 dilutions by ELISA. The results represent the average of three separate experiments (n = 4 mice in each three experiments) ± SEM. A. Anti-*E. coli *Ab response, B. Anti-*S. aureus *Ab response. Significant increase of antibody response was observed in the sera of mice after the third immunization (P < 0.05).

Sera of immunized mice collected over the course of three immunizations were analyzed by ELISA to determine the induction and duration of antibody response. Low but detectable serum antibodies specific for E. coli were found at comparable levels in all mice immunized with adjuvants after the second immunization. However, compared to mice immunized with E. coli alone (in PBS), those immunized with IFA, phytol, and PHIS-01 registered significantly high antibody levels (n = 5, P < 0.05) after the 3^rd ^immunization (Fig 1A). By contrast, in the case of S. aureus-immunized mice, only phytol, but not IFA or PHIS-01, engendered higher serum antibody response (n = 5, P < 0.05) after the 3^rd ^immunization (Figure 1B).

### Efficacy of adjuvant in sustaining antibody response induced

**Figure 2:**
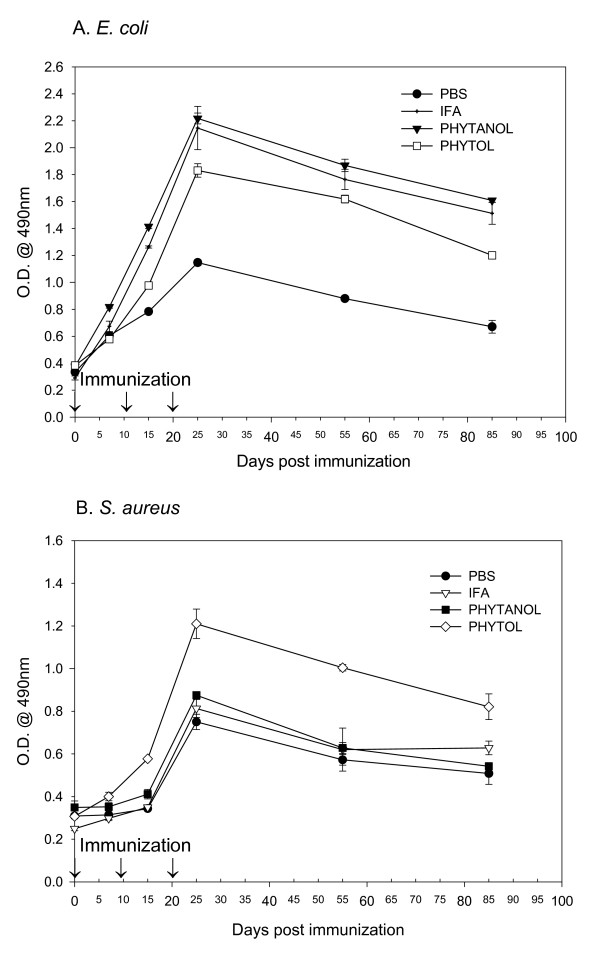
Efficacy of adjuvants in sustaining antibody response induced. BALB/c mice in groups of 4 or more were immunized at day 0, 10, 20 with bacterial vaccine in various adjuvants, and the antibody responses specific to either *E. coli *(A), or *S. aureus *(B) were determined by ELISA 30 and 60 days following the 3^rd ^immunization. Results are expressed as mean ± SEM.

The durability of antibody response elicited due to inclusion of adjuvants in the vaccine was determined 30 and 60 days after the third immunization. Although all mice receiving bacterial vaccines plus adjuvants evoked significant antibody response, its durability depended on the selection and inclusion of proper adjuvants. The results in Fig. 2A and 2B show that there was no steep decline in antibody level of mice immunized with either E. *coli *or S. *aureus *over a period of almost three months. It is noteworthy that both PHIS-01 and phytol were as effective as the standard adjuvant IFA in the case of E. *coli*, but for Gram-positive S. *aureus*, only phytol was more effective in providing sustained anti-bacterial antibody response.

### Isotype profile of strain-specific antibody

**Figure 3:**
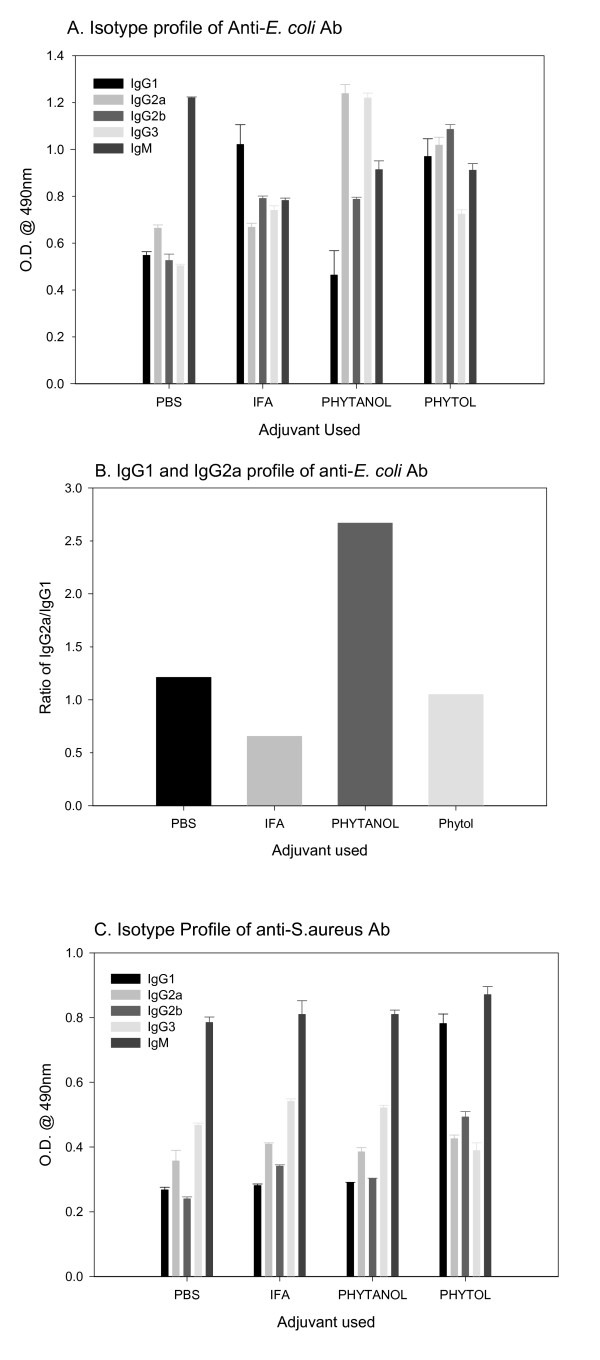
Isotypic profiles of humoral immune responses in mice immunized with bacterial vaccine formulated in different adjuvants. Sera for this assay were diluted 1:200 prior to detection of each isotype. Results are expressed as mean ± SEM. A. Isotypic profiles of anti-*E. coli antibodies*. B. Relative levels of IgG1 and IgG2a anti-E. *coli antibodies *C. Isotypic profiles of anti-*S. aureus *antibodies

IgG subclasses not only have relatively longer half-lives, they are also important in view of their specific effector functions. Therefore, we determined antibody isotype elicited in response to different vaccine formulations by ELISA using commercial, calibrated class-specific anti-sera. Whereas, the sera from mice immunized with *E. coli *in IFA and phytol showed high concentrations of IgG1, the IgG2a and IgG3 levels were higher only in PHIS-01-treated mice (Fig 3A). Notably, the ratio of IgG2a to IgG1 was >2.5 times higher only in mice immunized with PHIS-01 (Fig. 3B). In contrast, IgM was the predominant isotype in mice immunized with *S. aureus *and IFA or PHIS-01. However, only phytol and PHIS-01 elicited elevated levels of IgG1 in response to vaccination with *S. aureus *(Fig 3C).

### Antigenic specificity of serum antibodies

**Figure 4:**
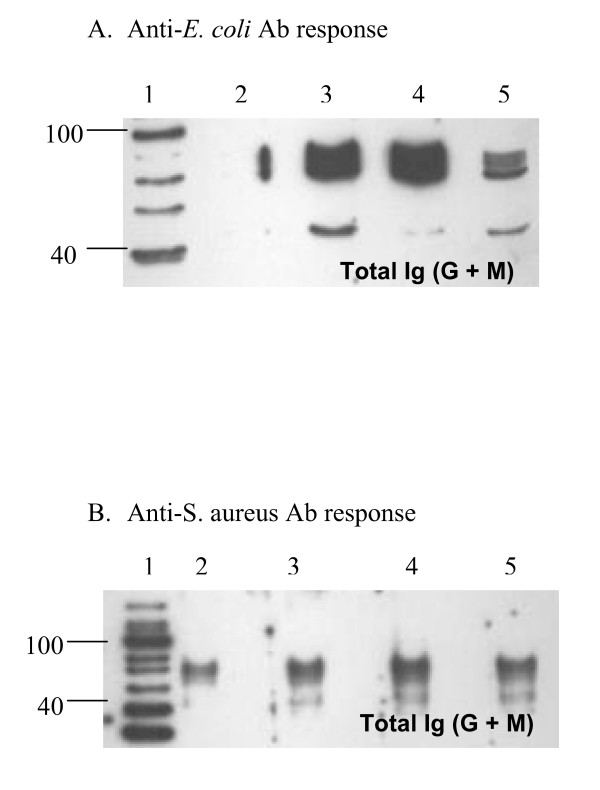
Western blot analyses of antigens recognized in bacterial lysates by mouse anti-sera induced with bacterial vaccine prepared in various adjuvants. Lane 1: Molecular marker; Lane 2: PBS (vaccinated with no adjuvant); Lane 3: IFA; Lane 4: PHIS-01; Lane 5: Phytol. Unimmunized controls or normal mouse sera revealed no protein recognizable by either antiserum. A. Antigens revealed by antisera from the *E. coli*-vaccinated groups. B. Antigens revealed by antisera from the *S. aureus *-vaccinated groups.

Figure 4 shows western blot analysis of serum samples obtained from mice immunized with heat-inactivated bacteria (60°C for 1 hr in a water bath) in various adjuvants. Antisera, used at 1:200 dilution during assay, recognized in *E. coli*, a few antigens of molecular sizes ranging from 40–100 KDa (Fig. 4A). The antisera in response to *S. aureus *recognized proteins of 45, 74, 87, 90 and 95 KDa (Fig 4B). It appears that anti-sera developed in response to *E. coli *using IFA and PHIS-01 were stronger than those obtained from mice immunized with phytol as adjuvant (Fig. 4A).

### Bacterial clearance from mice immunized with various adjuvants

Mice were challenged intraperitoneally with three doses (10^6^, 10^7^, or 10^8 ^CFU) of viable *E. coli *and *S. aureus*. Bacterial growth was determined in the peritoneal lavages harvested 18 and 36 hours after challenge with live bacteria. It was observed that when the challenge dose of either *E. coli *or *S*. *aureus *was 10^6 ^CFU, no bacteria was detectable in the control or vaccinated mice suggesting that they were eliminated by 36 hour from the blood streams and peritoneal fluids (Table 1 and Table 2). However, if the PBS and IFA-treated groups were infected with 10^7 ^CFU of *E. coli*, a large number of the bacteria were detectable in the peritoneal lavages even after 36 hours after the challenge, and importantly, no mouse survived the challenge with 10^8 ^CFU of the bacteria (Table 1). A striking contrast is readily apparent in the PHIS-01 or phytol-treated groups of mice since mice in both cases had no detectable *E. coli *after 36 hr (Table 1).

**Table 1:** Recovery of *E. coli *from peritoneal fluid obtained from mice challenged with live *E. coli*

Bacterial challenge (CFU)	10^6^	10^7^		10^8^	
Time	36 hrs post infection	18 hrs post infection	36 hrs post infection	18 hrs post infection	36 hrs post infection

^1^Control group (non-immunized)	0	^2^N/A	^2^N/A	^2^N/A	^2^N/A
^1^PBS	0	3.2 ± 1.1 × 10^4^	4.4 ± 1.2 × 10^5^	^2^N/A	^2^N/A
^1^IFA	0	1.8 ± 0.5 × 10^4^	6.0 ± 1.6 × 10^4^	^2^N/A	^2^N/A
^1^PHIS-01	0	0	0	1.1 ± 0.1 × 10^4^	0
^1^PHYTOL	0	0	0	2.1 ± 0.5 × 10^4^	0

^1 ^Peritoneal fluid samples from all groups of mice were diluted in LB medium (1:200), and 10 μl of diluted samples were streaked on agar plates using calibrated loops. CFUs were scored 18 and 36 hours following bacterial challenge and recorded as CFU/ml. The results represent the average of two experiments (n = 4 for each group) ± SEM. ^2 ^N/A: Not assessable since all mice were dead within 18 hrs.

**Table 2:** Recovery of *S. aureus *from peritoneal fluid obtained from mice challegened with live *S. aureus*

Bacterial challenge (CFU)	10^6^	10^7^		10^8^	
Time	36 hrs post infection	18 hrs post infection	36 hrs post infection	18 hrs post infection	36 hrs post infection

Control group (non-immunized)	0	1.08 ± 0.14 × 10^6^	8.98 ± 1.94 × 10^6^	1.11 ± 0.20 × 10^8^	^2^N/A
PBS	0	4.92 ± 0.94 × 10^5^	1.54 ± 0.94 × 10^6^	3.16 ± 1.94 × 10^6^	^2^N/A
IFA	0	2.35 ± 1.12 × 10^5^	1.23 ± 1.14 × 10^6^	1.80 ± 0.22 × 10^6^	2.16 ± 1.10 × 10^6^
PHIS-01	0	3.64 ± 1.44 × 10^5^	1.13 ± 2.31 × 10^6^	8.90 ± 3.20 × 10^5^	5.60 ± 0.94 × 10^5^
PHYTOL	0	4.0 ± 0.25 × 10^3^	0	8.90 ± 1.12 × 10^4^	0

^1 ^Peritoneal fluid samples from all groups of mice were diluted in LB medium (1:200), and 10 μl of diluted samples were streaked on agar plates using calibrated loops. CFUs were scored 18 and 36 hours following bacterial challenge and recorded as CFU/ml. The results represent the average of two experiments (n = 4 for each group) ± SEM. ^2 ^N/A: Not assessable since all mice were dead within 18 hrs.

In the case of *S. aureus infection also*, the challenges with higher than 10^6 ^CFU caused a considerable number of the bacteria to persist in the peritoneal fluids of mice treated with PBS, IFA and PHIS-01. The unimmunized control and the PBS-treated vaccinated groups succumbed to infection and died within 36 hrs after challenge. The IFA and PHIS-01 groups survived during this period and eventually the PHIS-01-treated mice survived. Most noteworthy, however, is the effect of Phytol, which registered no bacterial detectable growth 36 hrs after challenge and resisted infection most effectively (Table 2).

### Influence of adjuvants on survival from bacterial challenge

**Figure 5:**
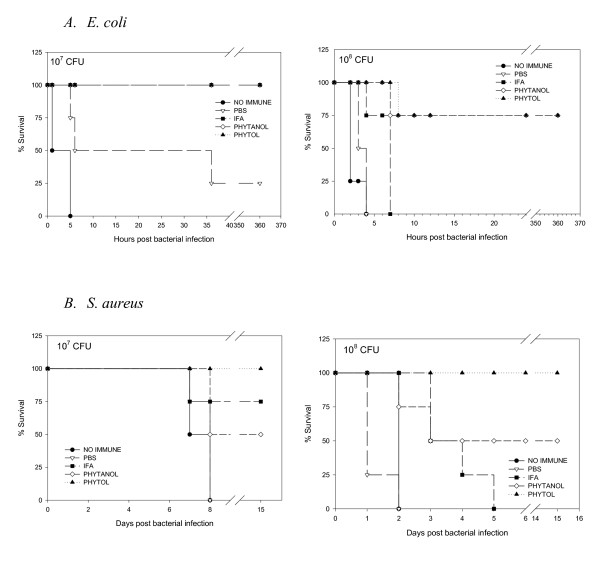
A. Survival from *E. coli *infection. B. Survival from *S.aureus *infection. Each group of ten mice immunized with bacterial vaccine formulated in various adjuvants was challenged with live bacteria (10^6^, 10^7^, and 10^8^), and observed for survival.

To examine whether adjuvants differ in effectiveness in terms of animal survival, mice were inoculated with 10^6^, 10^7^, or 10^8 ^CFU per mouse of *E. coli *or *S. aureus*. As shown in Figure 5 (A&B), mice immunized with 10^6 ^CFU of either *E. coli *or *S. aureus *showed few symptoms and all mice survived. However, mice challenged with larger inocula such as 10^7^, or 10^8 ^CFU of either bacterium were lethargic and suffered from loose stools. If death did not occur within 24 hrs of bacterial challenge, the mice survived from the infection. Phytol and PHIS-01 were both much more effective than PBS (i.e., vaccines with no adjuvants) and IFA in conferring protection against *E. coli*. Interestingly, the overall survival rate of mice immunized with *S. aureus *was somewhat higher than in mice immunized with *E. coli*. Mice vaccinated with *S. aureus *and phytol showed the best protection against *S. aureus *challenge.

### Effects of adjuvants on inflammatory cytokines (IL-6 and TNF-α)

**Figure 6:**
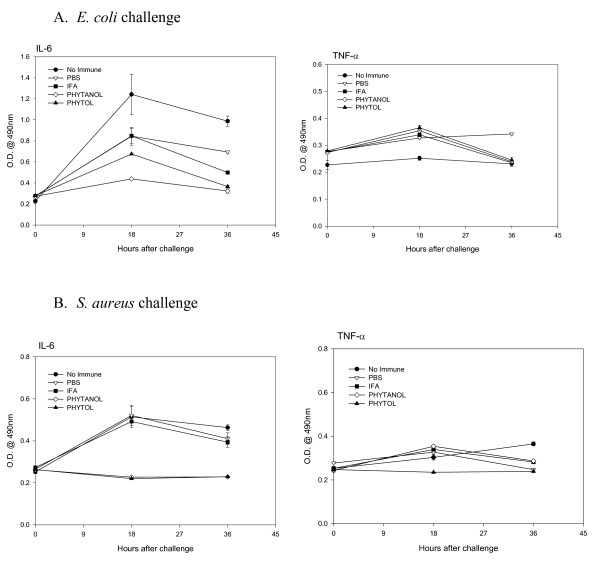
Effect of adjuvants on proinflammatory cytokine production (IL-6 and TNF-α) in response to bacterial vaccine. The levels of IL-6 and TNF-α were determined by ELISA in peritoneal lavages obtained from mice immunized with bacterial vaccines followed by challenge with either live *E. coli *(A), or *S. aureus *(B).

In order to determine whether adjuvants exert any effects on induction of inflammatory cytokines, such as IL-6 and/or TNF-α, the levels of these cytokines were measured in peritoneal lavages. The results obtained from mice challenged with 10^6 ^CFU of *E. coli*, or *S. aureus *are shown in Fig. 6. Since bacterial inocula larger than 10^6 ^CFU killed all control unimmunized mice within 24 hrs, these experiments were performed using only 10^6 ^CFU of each bacterium. There was no significant increase in the TNFα-level in all groups of mice, whereas the IL-6-level was significantly lower in phytol and PHIS-01-treated mice inoculated with *E. coli *(Fig 6A). In contrast, there was much less of either cytokine induced during the *S*. *aureus *challenge (Fig 6B).

## Discussion

There has been, to our knowledge, no prior systematic investigation involving the efficacy of dietary isoprenoids, such as phytol or phytol-derived compounds, as adjuvants. In our ongoing study [12], we have tested the adjuvant activity of phytol, and its reduced derivative, PHIS-01, and observed efficient stimulation of antibody response against hapten antigens conjugated to a protein carrier. We have observed that the response due to these adjuvants appeared superior to what has been observed with conventional adjuvants, such as CFA/IFA, TiterMax, and Alum. PHIS-01, in particular, also efficiently evokes cellular immunity, including tumor-specific cytotoxic and helper T cell responses [12]. In this report, we have assessed the usefulness of the adjuvant potentials of phytol and PHIS-01 in augmenting efficacy of vaccines against the common infectious agents *S. aureus *and *E. coli*.

Our vaccine formulations contain heat-inactivated bacteria emulsified with standard IFA, or either of the two experimental phytol-based adjuvants. The latter, unlike IFA, have been used without any emulsifying or surface-active agents. In spite of these differences, these new adjuvants are effective not only in augmenting anti-bacterial humoral responses against both *E. coli *and *S. aureus*, but also in preventing bacteremia and death caused by these infections. Phytol and its derivative seem to be excellent adjuvants for their ability to enhance and sustain quality antibody responses (preventing bacteremia) over a longer period of time. Thus, phytol-based novel adjuvants significantly improve vaccine efficacy by modulating immunogenicity and toxicity of the heat-killed bacterial inocula, responsible for gram-negative bacteremia [16-18]. However, phytol and PHIS-01 adjuvants differ in their effectiveness against gram-positive *S. aureus*. Phytol is better at increasing specific antibody responses and preventing bacteremia and death due to *S. aureus*.

It has been well known that IgG2a is the most desirable antibody isotype for therapeutic applications involving normal immune responses [19]. This isotype is more effective in activating complement, promoting antibody-dependent cellular cytotoxicity, and conferring protection against tumors or parasite invasion than any other isotype. In mice immunized with *E. coli*, phytol and in particular, PHIS-01 exert their effects in raising mouse serum levels of all major IgG subclasses, specifically IgG2a antibody. In contrast, mice vaccinated with *S. aureus *lysates emulsified with phytol register higher levels of IgG1-type antibody, and are better protected, whereas IFA and PHIS-01 do not exert much effect on this isotype switch. Both IFA and PHIS-01 promote induction primarily anti-staph IgM response, which is not associated with the immunological memory. This induction of IgG1 antibody against gram-positive *S. aureus *observed with phytol implicates Th2-type cellular responses and the establishment of immunological memory.

Adjuvants facilitate the persistence of antigens at injection sites, the so-called depot effect. The qualitative differences in adjuvant efficacy can also be gleaned from the analyses of antigens involved in immune responses. The *E. coli *antigens recognized by immune sera due to phytol and PHIS-01 are clearly discernible on western blots as compared to immune sera obtained from IFA-immunized mice. A 45 KDa antigen was recognized by antibodies from mice immunized with phytol and PHIS-01 only. Similarly, IgG antibodies only from the phytol group recognized four unique *S. aureus *antigens (approximately 45, 74, 90 and 95 KDa). Our findings suggest that phytol and PHIS-01 differ from the conventional adjuvant IFA in their ability to augment the immunogenicity of bacterial antigens. This may explain why phytol and its derivative provide better protection against re-exposure to the pathogens. The biochemical nature of these antigens remains to be elucidated.

The efficacy of phytol and PHIS-01 as adjuvants is also evident in the quality of protection that the vaccines provide against infection from the early onset. In lethal cases, a marked fall in antibody levels invariably increases the probability of high mortality in mice [20]. Mice immunized with killed *E. coli *and phytol or PHIS-01 showed transient bacteremias and all survived. The state of immunity was maintained even when mice were challenged with 10^6 ^CFU of *E. coli *cells. These important results suggest that the immunological memory against relevant antigen(s) can facilitate rapid elimination of the infectious agent from the blood streams and peritoneum. In contrast, mice immunized with either PBS or IFA die within 1–3 days.

Underlying mechanisms defining the differences in adjuvanticity of phytol and PHIS-01 relative to IFA could potentially lie in monocytes and macrophages that produce pro-inflammatory cytokines such as TNF-α and IL-6. These cytokines play important roles as mediators of fatal septic shock [22-25]. In our investigation, little TNF-α could be detected at 18 hr after bacterial infection. However, IL-6 is detectable in peritoneal lavage and blood for a short period in mice immunized with PBS and IFA, but not phytol and PHIS-01. It is apparent from this study that heat-inactivated microorganisms are not immunogenic enough to induce an effective immune response, and that adjuvants in vaccine formulations could make the difference. Our results demonstrate the usefulness of phytol and PHIS-01 as effective adjuvants. Interestingly, mice that survive bacterial infections produce less TNF-α and IL-6 cytokines, the important mediators of fatal septic shock.

Thus, the development of muti-epitopic *E. coli *and *S. aureus *vaccines using these adjuvants appears promising. The other area of interest is to develop anti-bacterial gamma globulins for intravenous use preventing toxemic episodes. It has previously been shown by Kaijser et al. that the passive administration of a monoclonal Ab specific to *E. coli *in conjunction with an antibiotic significantly improves the survival of animals with experimental infection [26]. High-risk patients with poor host defense, such as prematurely born infants and patients undergoing immunosuppressive chemotherapy, may even benefit from passive infusion of immune responses induced in competent individuals. Since most fatal nosocomial infection is caused by *E. coli *and *S. aureus, *it will be of interest to develop IgG-enriched vaccines for prophylaxis and for the treatment of nosocomial sepsis.
